# Impact of sediment parameters in the prediction of benthic microbial fuel cell performance†

**DOI:** 10.1039/d0ra03459b

**Published:** 2020-07-10

**Authors:** Kevin L. Joiner, Gabriel L. Tukeman, Anna Y. Obraztsova, Yolanda Meriah Arias-Thode

**Affiliations:** Naval Information Warfare Center San Diego USA kevin.l.joiner1@navy.mil; Baylor College of Medicine Houston Texas USA

## Abstract

The benthic microbial fuel cell (BMFC) is a promising technology for harvesting renewable energy from marine littoral environments. The scientific community has researched BMFC technology for well over a decade, but the *in situ* performance remains challenging. To address this challenge, BMFC power experiments were performed on sediment collected from San Diego Bay (CA, USA), La Spezia (Italy) and Honolulu (HI, USA) in the ever-changing littoral environment. Analysis of BMFC laboratory data found the power density varied substantially across 11 sites in San Diego Bay. In addition, data from experiments repeated at four locations in San Diego Bay showed significant differences between experiments performed in 2014, 2016 and 2019. Multivariable linear analysis showed BMFC 90 day cumulative power density was positively correlated with the total organic carbon (*p* < 0.05) and negatively correlated with the black carbon in the sediment (*p* < 0.05). Regression coefficients trained on the San Diego Bay data from 2014 facilitated accurate predictions of BMFC performance in 2016 and 2019. The modeling paradigm accurately explained variations in BMFC power performance in La Spezia and showed sediment parameters can impact BMFC performance differently across geographic regions. The results demonstrate a great potential to use sediment parameters and statistical modeling to predict BMFC power performance prior to deployment in oceanographic environments, thereby reducing cost, work force and resources.

## Abstract 2

BMFC power output is correlated with costal sediment parameters to predict BMFC performance in the natural environment.

## Graphical abstract

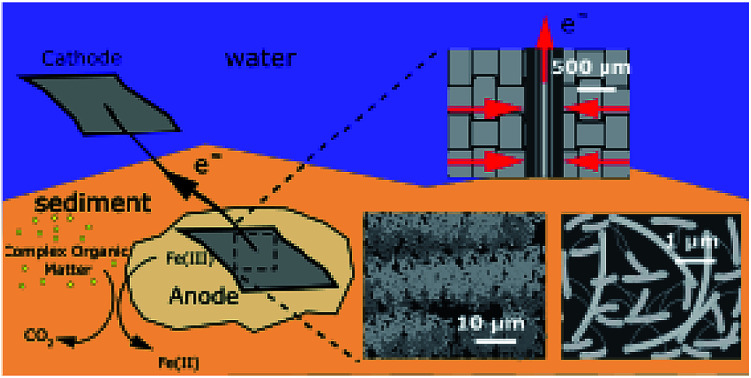

## 1 Introduction

Traditionally, oceanographic devices and sensors are powered *via* battery or similar sources, that become depleted and need constant replacement, maintenance, or are discarded in place. Benthic microbial fuel cells (BMFCs) are nature friendly, low maintenance, bioelectrochemical systems for harvesting continuous electrical power from marine environments.^1–5^ Though the amount of power produced by BMFCs in the field is on the order of 0.1–0.5 watts,^3,6^ recent field deployments have demonstrated their ability to sustain low-power oceanographic equipment, either as a sole energy source or by supplementing traditional power sources, with little or no maintenance.^6^ This increased practicality of BMFCs has increased the need to know their potential power performance prior to field deployment by identifying and quantifying certain site-specific sediment qualities that optimize their power output.^2,7–10^ While BMFCs are desirable alternative power sources for low-power marine devices and sensors,^1,3,4,8,11^ their *in situ* predictability presents a challenge.

During the harvesting process, naturally occurring anaerobic microorganisms in sediment convert chemical energy into electrical energy through oxidation and reduction reactions.^1,9,12,13^ These catalytic reactions are facilitated with electrically connected anodes, which are buried in sediment, and cathodes, which are suspended in the overlying water column. Microbes at the anode metabolize organic substrates and electrons are transferred to oxygen, which serve as the terminal electron acceptor at the cathode. Finally, current production is sustained in these benthic environments *via* a constant influx of replacement nutrients, supplying electrons by way of natural water flow, often through pore water.^14^

Microbial fuel cell field performance in marine environments has been researched for approximately two decades, but most studies have focused on fairly isolated geographic areas.^2,3,8–11,15,16^ For example, in more classical studies, Reimers *et al.* (2001)^8^ simulated the marine environment in a laboratory using sediments extracted from a salt marsh near Tuckerton (NJ, USA) and an estuarine site within Raritan Bay (NJ, USA) to show microbial fuel cells could power oceanographic instruments deployed for long-term monitoring. Tender *et al.* (2002)^10^ deployed two SMFCs in coastal marine environments, in Tuckerton, and Yaquina Bay Estuary (OR, USA) to observe the potential gradient between an anode buried in marine sediment and a cathode in overlying seawater and show the possibility of power generation from marine sediment. Reimers *et al.* (2006) showed that a cold seep has the potential to provide more power than neighboring ocean sediments in Monterey Canyon (CA, USA).^11^ Donovan *et al.* (2008) deployed a SMFC in the Palouse River (WA, USA) to power a wireless temperature sensor^2^ and Tender *et al.* (2008) demonstrated MFCs can power sonobuoys located in the Potomac River, Washington D.C and a salt marsh near Tuckerton.^3^ In more recent and relevant studies, Zhao *et al.* (2016) collected sediment from Horseshoe Lake (North China) to investigate how variation in organic matter loading affected electricity generation in SMFCs^15^ and Kubota *et al.* (2019) operated a set of five SMFC's at a single site on the seafloor in Tokyo Bay, to evaluate their electrochemical characteristics and effects of sediment on the anode.^16^ Such geographically confined case studies may vastly misrepresent MFC performance across large regions as microbial communities may shift or adapt due to varying substrate distributions or other environmental pressures. This is crucial, as locations with different sediment quality may yield varying results in sediment based MFC power leading to either the success or failure of their desired application.

One means of determining sediment quality is by measuring the total organic carbon (TOC) present in the sediment.^17^ Under relatively dry conditions, soil/sediment minerals act as adsorbents of organic matter, where sorbed organic compounds are held on the minerals surface.^17^ Previous studies have shown some amount of positive correlation between sediment TOC concentration and power production in microbial fuel cells.^11,18^ However, there have been few studies demonstrating how or if total organic carbon, either solely or in combination with other predictors, can be used to efficiency predict BMFC power production. Another means of determining sediment quality, is by measuring its amount of black carbon (BC)—the carbonaceous residue of incomplete combustion of organic matter and includes compounds such as charred biomass and soot.^19^ Numerous studies of sediment carbon have demonstrated the ubiquitous presence of BC in the environment.^19–21^ For instance, black carbon is deposited into marine sediments *via* rivers and (or) atmospheric transport mechanisms.^20^ Previous studies have shown that BC accounts for 4–22% of marine dissolved organic matter and 9% of TOC in sediments worldwide.^19,21^ These compounds are resistant to thermal or chemical degradation, and their resistivity to breakdown makes BC representative of a carbon sink in the environment.^22–24^ Additionally, BC is resistant to microbial breakdown preventing its use as a nutrient source in the case of BMFC's.^22^ Given the promising potential of BMFC's as an alternative energy source and the relatively unknown effect of BC on BMFC power production, this makes the impact of BC on BMFC power production an important and interesting topic for study.

**Fig. 1:**
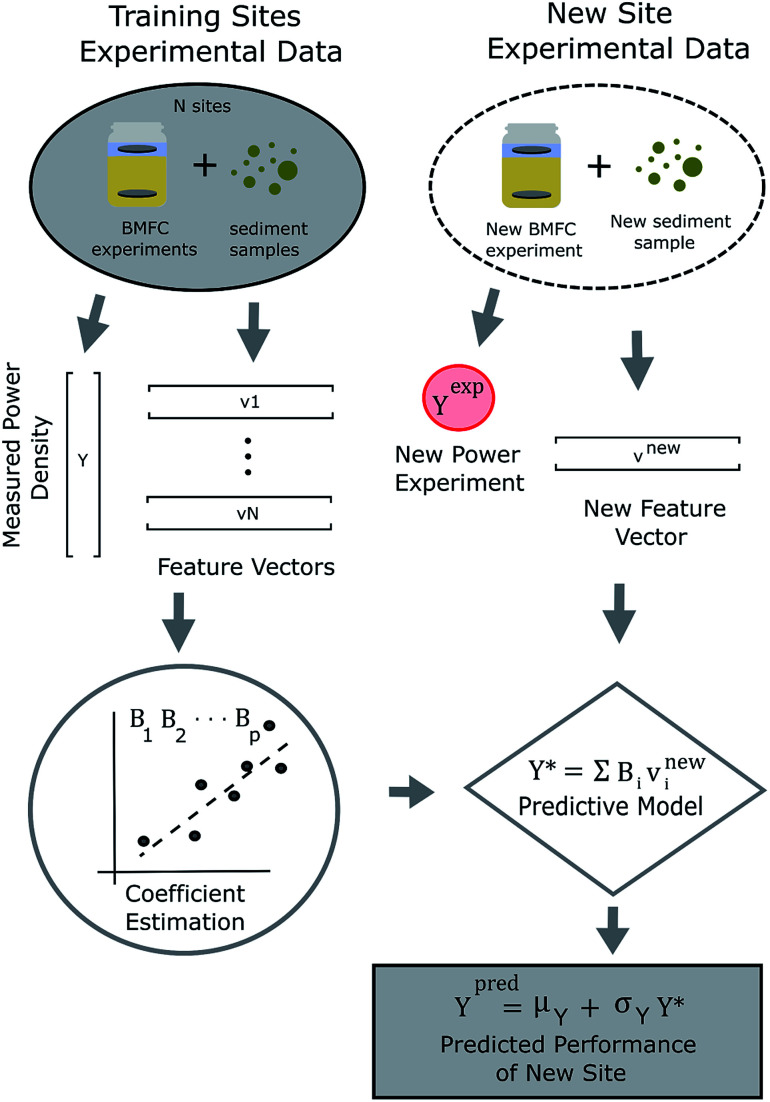
Process flow of a hypothetical statistical modeling process for BMFC power prediction.

**Fig. 2:**
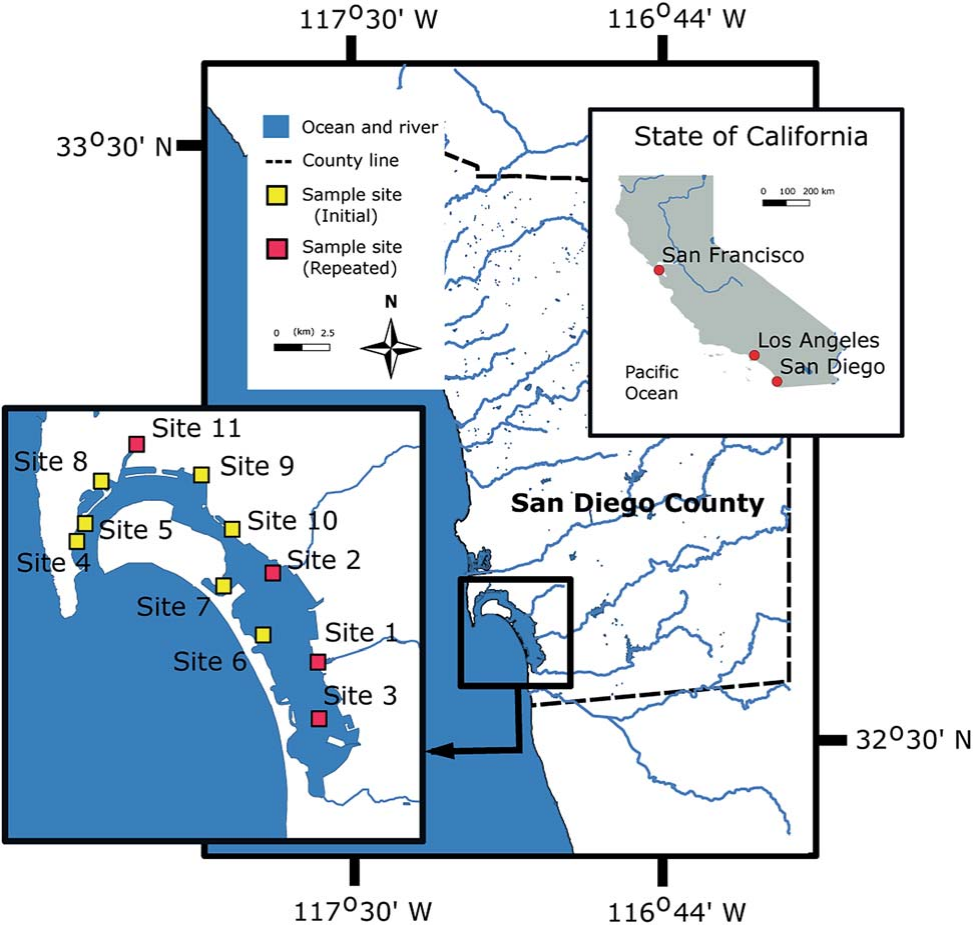
Location of sediment sampling in San Diego Bay. Multiple samples were collected at all locations. See the ESI Table S.1† for precise locations. Sites labeled as “repeated” denote BMFC experiments and sediment analyses performed three years after those labeled as “Initial”.

We hypothesize BMFC power performance can be forecasted using sediment characteristics (such as TOC, BC, C : N and grain size) as predictors. To test this hypothesis, a relatively simple statistical learning process for predicting BMFC power performance was conceived and its schematic is shown in Fig. 1. For the process, feature vectors of sediment characteristics from sites near one another were used to described larger geographic regions in a quantitative manner. A generalized statistical model, using tunable parameters (an array of *B*'s), makes predictions on BMFC performance at new sites within a region. For this study, the process was put into practice using sediment samples collected from San Diego Bay (see Fig. 2) that provided natural fuel for BMFC power density experiments in the laboratory. A multivariable linear regression model was constructed and trained on the BMFC power density response to TOC and BC sediment features in local sediment. The model was then used to predict BMFC performance for experiments repeated two and three years later for four sites in San Diego Bay. Training the model on sediment and BMFC power performance data from four sediment samples from La Spezia (Italy) and three sediment samples from Honolulu (HI, USA) tested the robustness of modeling parameters outside the San Diego region. The predictors relative dispersion and a measure of their contribution to the predicted BMFC power performance response provided insight into possible physical mechanisms behind long-term BMFC performance in different sediment conditions.

## 2 Experimental

### 2.1 Station sediment sampling and preparation for analyses

Sediments were collected from eleven locations in San Diego Bay (CA, USA) (Fig. 2) and two sites in La Spezia (Italy) (see Table S1† for exact locations) during sampling campaigns conducted between 2014 and 2016. Some sediment samples from the same locations in San Diego Bay and two additional sites in Honolulu (HI, USA) were obtained in 2019 to repeat sediment analyses and BMFC experiments. Sample collections were dictated by areas where BMFC systems were deployed. The collection depths of sediment were about 5 (San Diego Bay), 10 (La Spezia) and 12–14 m (Honolulu). The bulk of the sediment was scooped from approximately 20 cm into the surface with a Van Veen grab sampler, placed into glass jars and brought to the cold room (4 °C) to store until use.

**Fig. 3:**
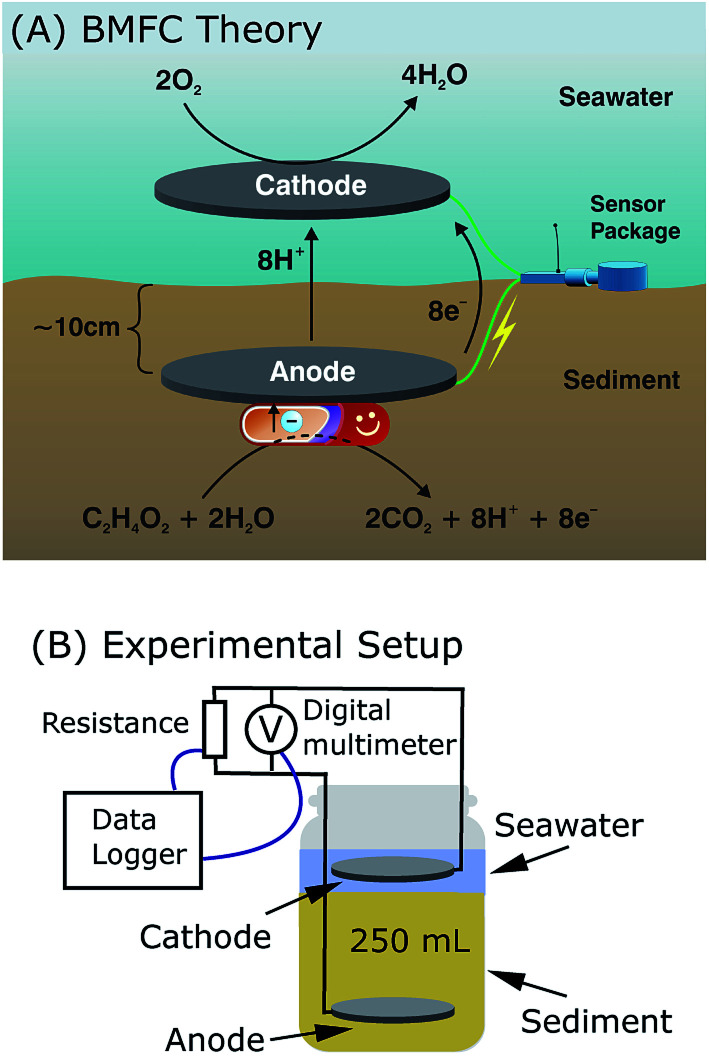
BMFC theory and experimental methodology. (A) Benthic microbial fuel cell theory as being applied to powering underwater sensors. (B) Experimental setup of BMFCs that replicate site environments under controlled conditions.

Sediment samples were analyzed at ALS (Australian Laboratory Services) Environmental (Tucson, AZ) for carbon, hydrogen and nitrogen (C : N ratio), total organic carbon (TOC), total black carbon, and grain size (for sand and silt percentages). In order to prepare sediments for analyses, approximately 1 kg of each sample were homogenized and large organic and non-organic fragments were removed. Then, duplicate samples (0.15 kg each) were sent for analysis to ALS Environmental where sediments were lightly ground and split into sub-samples, one for particle size and one for elemental analyses. Wet sediment samples were air dried at 40 °C for several days and measured for moisture loss to determine water content. Afterwards, the sub-samples were ground to a fine powder. For grain size analysis, sediments were classified using a wet sieve analysis for cementation of sediment particles. Samples were mixed with a 4% sodium hexametaphosphate dispersing agent and allowed to soak for 16 hours. This slurry solution was then blended with deionized rinse water at 10 000 RPM for one minute prior to sieving. The silt clay boundary was taken at 100 μm and sediment components that passed through were considered silt (per ALS Environmental procedures) (Fig. 3).

### 2.2 BMFC power production experiments

#### 2.2.1 Anode and cathode construction

Zoltec™ 40 carbon fiber cloth was used for both anode and cathode fabrication. Anodes were cut into 7 cm squares. Exposed titanium wire was woven through the cloth and secured using a zig–zag stitch along its length. Precise measurements of the anode sizes were recorded for calculation and normalization purposes. Anodes shared a common cathode, which was cut to 1.5 to 2 times the size of the combined anode surface areas. Solid titanium wire was woven through the length of the cathode and secured with a zig–zag stitch.

#### 2.2.2 BMFC mesocosm assembly

Mesocosms were assembled using 400 mL glass beakers (Pyrex) that housed each anode in the sediment. Sediment was filled approximately to the 250 mL mark and tapped to remove air pockets. Anodes were pushed into the sediment until completely covered. The beakers were then filled with sand-filtered sea water from San Diego Bay, and the sediment was allowed to settle. The mesocosms were then placed in large plastic tanks with a water outlet system, with seven to nine units in each tank. The tanks were filled with sand-filtered sea water, with aerators used to oxygenate the water column and the water temperature was maintained at 15 °C. In comparison with the actual environment, the water temperature of San Diego Bay typically ranges between 14 °C in the winter months to 20 °C in late summer. The BMFC systems were left in open circuit with flow-through conditions until a reference voltage difference between the anode and the cathode was 0.7 V to 0.8 V was reached. Cultured bacteria were not introduced into the fuel cells, but instead indigenous microbial communities were allowed to develop from the sediment or seawater.

#### 2.2.3 MFC regulation and monitoring

In the preliminary experiments of the 11 sites in San Diego Bay, the anodes and cathodes of each BMFC were electrically connected *via* a terminal block with titanium wires. The BMFCs were maintained at 0.4 V using resistors, and were manually charged once daily for the duration of the experiment. The resistor value, cathode–anode voltage, and anode size were used to calculate power and power density.

In all subsequent experiments, MadgeTech™ 8 Channel ± 100 mV data loggers were used to collect current measurements of each BMFC system. Customized potentiostat boards^25^ were fitted with 1000 Ω, 499 Ω, 100 Ω, and 49.9 Ω resisters to ensure that the fuel cells were held at 0.4 V while remaining within the reading-frame of the MadgeTech™, which was −100 mV to 100 mV. Each potentiostat board was powered by a 9 V battery, which was changed when needed. Electronics were housed within a 2.9 gallon leak-proof container with a desiccant to remove excess moisture. The cathode–anode voltage was recorded manually every day for power output calculations. The MadgeTech data loggers were programmed to record voltage measurements once every five minutes for a sample rate of 12 times per hour. The data was downloaded every few days to track progress and determine the need for battery or resistor replacement.

### 2.3 Statistical analysis and modeling

#### 2.3.1 Statistical analysis of BMFC power density

For the study, BMFC performance was measured in terms of its power density (electric power divided by anode surface area) over time. Data downloaded from the MadgeTech™ loggers were exported to Microsoft Excel and run through a custom MATLAB program to calculate values for BMFC power density, *P*_d_(*t*), sampled at times (*t*) that were separated by approximately one week intervals Δ*t* (≈7 days). To assess each site's power performance at time *t*, the average power density, 
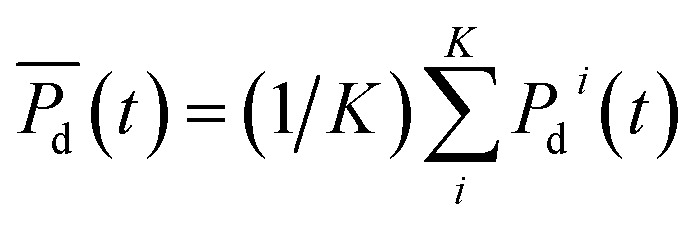
, was calculated over *i* = 1, …, *K* replicate experiments. Summing the average power densities together and dividing by the total number of sampling weeks, *W*, gave each sites cumulative average power density: 
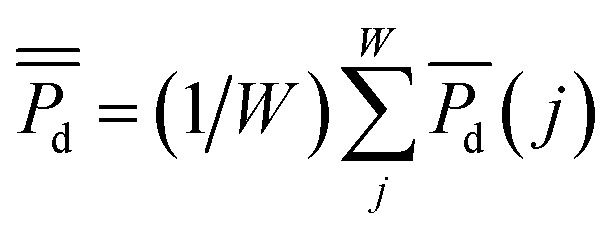
 for *W*Δ*t* days. For example, if *W* = 13 weeks then *W*Δ*t* = 90 days. Independent *t*-tests were used to determine any difference between power density curves. Two way mixed analysis of variance (ANOVA) was used to test for significant correlations between cumulative average power densities and sediment qualities (carbon concentrations and grain size). The significance level of the *p*-values for all tests was set at *p* ≤ 0.05.

#### 2.3.2 Multiple linear regression modeling

The multiple linear regression model of a particular population with *p* predictors (*X*_1_, *X*_2_, …, *X*_*p*_), (TOC, BC, C : N, grain size) and one dependent variable 
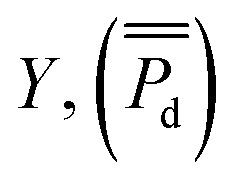
 was specified as
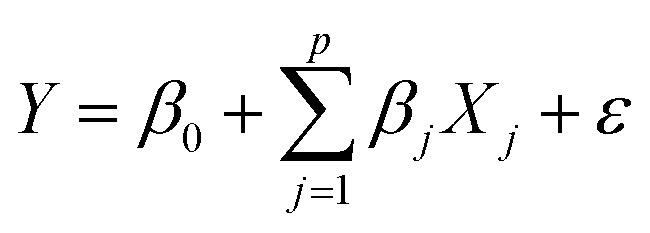
where *β*_*j*_ was the regression coefficient associated with the *j*th predictor, *X*_*j*_, and *ε* was the random error with variance *σ*^2^ and zero mean.^26^ A randomly selected set of *n* observations from a given region consisted of the set {*y*_*i*_, *x*_*i*1_, *x*_*i*2_, …, *x*_*ip*_}, *i* = 1, 2, …, *n* where each entry represented the *i*th sites response (*y*_*i*_) to the predictors (*x*_*i*1_, *x*_*i*2_, …, *x*_*ip*_). Using this data set, the response and predictor variables where expressed in a standardized form, having zero mean and vector length one, *via* the formulas1
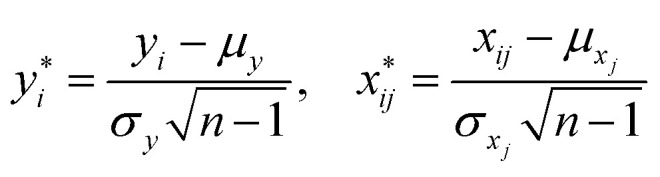
where *μ*_*y*,*xj*_ and *σ*_*y*,*xj*_ were the sample means and standard deviations of the response and predictors respectively. Using this standardization, un-weighted least squares was employed to obtain the estimated linear model2
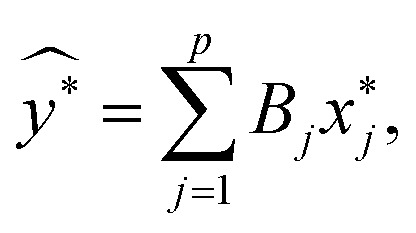
where the *B*_*j*_ was the standardized estimated regression coefficient associated with 
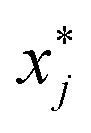
. Estimated responses (predictions) were computed as *ŷ* = *μ*_*y*_ + *σ*_*y*_y*.

#### 2.3.3 Measure of predictor impact

The general dominance index *D*_*j*_ is a statistic commonly used in multiple linear regression analysis.^27,28^ It is defined as the average increment in the coefficient of determination associated with predictor *x*_*j*_ across all possible sub-models. Let *D*^*k*^_*j*_ be the average increase in the coefficient of determination due to adding *x*_*j*_ to 
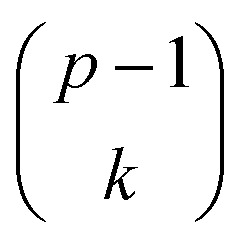
 different submodels each with *k* variables, *k* = 0, 1, …, (*p* − 1). Expressed mathematically,
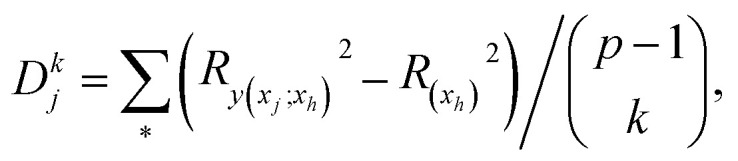
where *x*_*h*_ is any subset of *k* predictors with *x*_*j*_ excluded and 
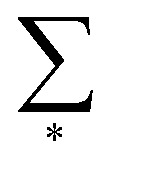
 denotes the sum over all 
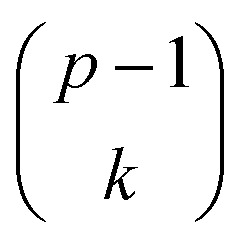
 submodels. The proportion of variance explained, *R*^2^ (abbreviated *R*_sq._), was calculated using the formula*R*^2^ = 1 − RSS/TSSwhere 
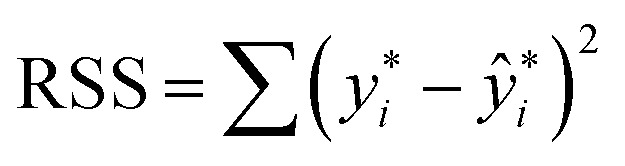
 and 
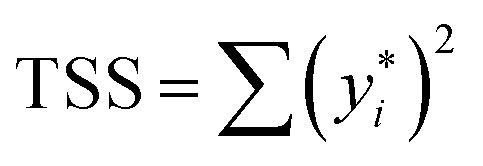
. Then, *D*_*j*_ is obtained by averaging over all *p* submodel sizes, namely;3
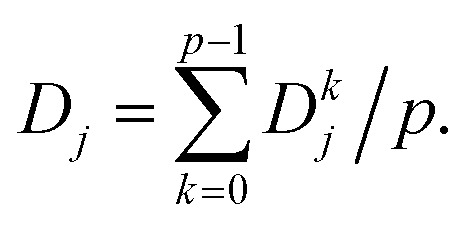


In this way, *D*_*j*_ offers a general framework for determination of relative impact of predictors in the linear multiple regression model.

## 3 Results and discussion

### 3.1 San Diego Bay 2014 experiments and modeling

**Fig. 4:**
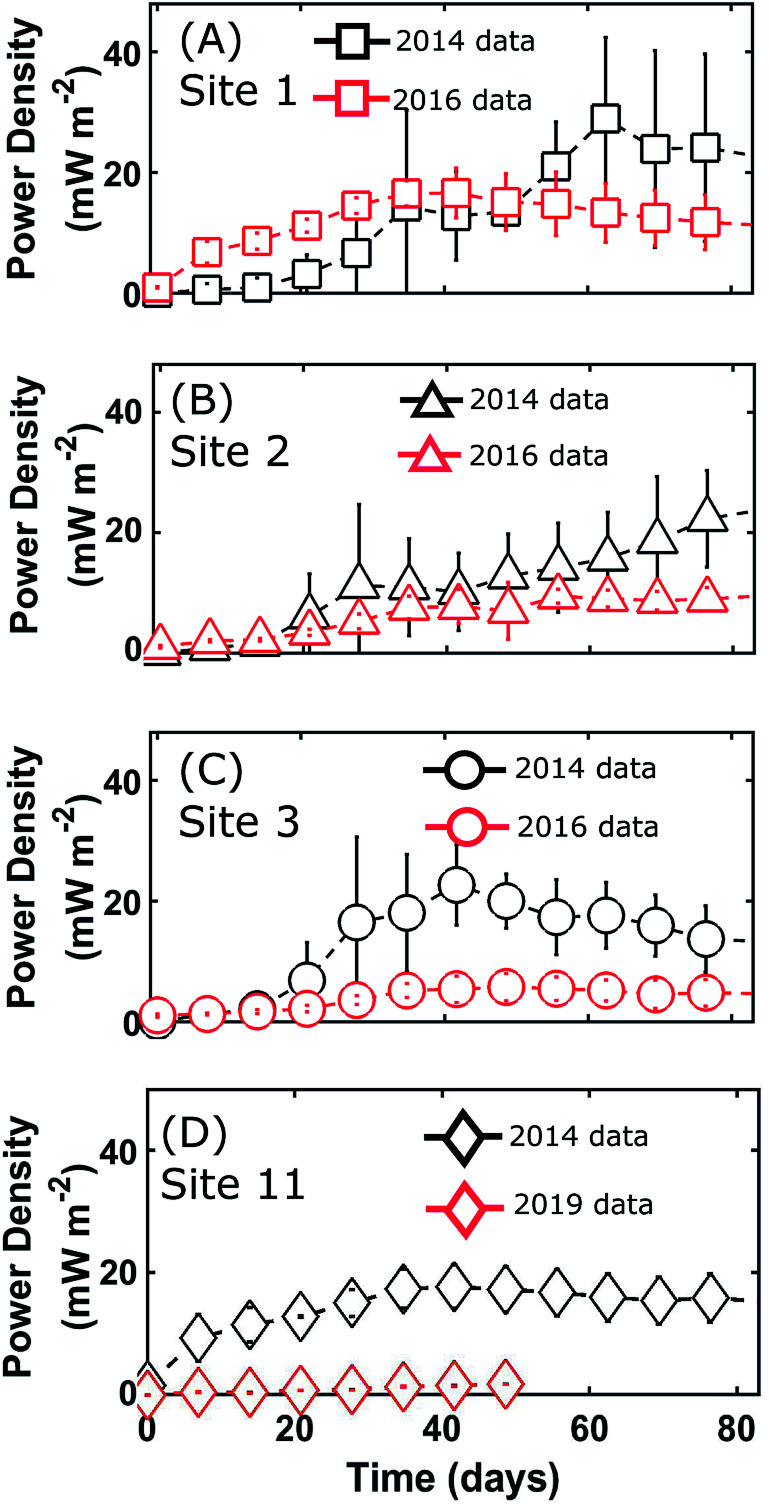
Comparing power density from initial and recurrent San Diego Bay BMFC experiments in sites 1 through 3 and site 11. Samples were taken in August 2014 (black), September 2016 (red) and September 2019 (red). Each time series represents average power densities 
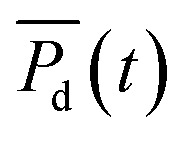
 (error bars ± SD) at sample time *t*. Symbols and error bars are shown at approximately one week intervals.

BMFC power experiments were performed on sediments from 11 sites around San Diego Bay in 2014 (Fig. 2). During these BMFC experiments, the power density curves for each of the 11 sites tended to begin with a lag-phase lasting anywhere from days to weeks, with a slight peak followed by either a steady or slowly decaying state (see Fig. 4 and ESI Fig. S.1†). These traits are typical of power density curves observed in BMFCs—during the startup phase, there is a sufficient supply of nutrient to the biofilm, but as nutrients are consumed over time, power production becomes dependent on a fresh supply of nutrient presumably through pore water. The cumulative average power densities, 
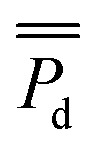
, of the lab experiments ranged from 0.38 to 14.0 mW m^−2^ (ESI Table S.2†), that were on the same order of average power densities found in previous studies of benthic microbial fuel cells^8,10,11,29^ (10–34 mW m^−2^). The BMFC population mean cumulative average power density from the San Diego Bay sediments was *μ*_*P*_d__ = 6.92 mW m^−2^ with standard deviation *σ*_*P*_d__ = 4.99. These results indicated BMFC power performance was not constant, but instead varied across the different sites in the San Diego Bay region.

Laboratory analysis revealed sediment quality was highly varied throughout San Diego Bay and its geochemical and textural properties is given in ESI Table S.2.† Carbon to nitrogen ratios varied near or around typical values (8 to 17) found in soil samples throughout the world.^30^ The sediment TOC content ranged from 0.15 to 1.96 (wt%). The extreme high values of TOC were found at site 11 where the sediment grain size was fairly fine at 94.1 (wt%) silt & clay. The lowest value of TOC was 0.15 (wt%) and found at site 10 which was composed of 11.4 (wt%) silt & clay with coarse, sandy sediment conditions. The BC content of sediment ranged from a low of 0.10 to a high of 0.25 (wt%) but did not appear to be well correlated with any other sediment characteristics. The sample means and standard deviations of TOC and BC where *μ*_TOC_ = 0.97 (wt%), *σ*_TOC_ = 0.60 and *μ*_BC_ = 0.18 (wt%), *σ*_BC_ = 0.05 respectively.

Testing different linear combination of the sediment parameters (TOC, BC, C : N and grain size) analyzed for this study revealed TOC and BC to be the most significant predictors in explaining the variance in BMFC cumulative average power densities across test samples. Thus, the final model was specified to be4
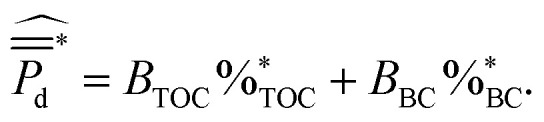


Ordinary least squares was applied using data on the 11 initial samples in San Diego Bay (ESI Table S.2†) giving the fitted parameters *B*_TOC_ = 1.55 and *B*_BC_ = −1.06. ANOVA statistics (ESI Table S.3†) revealed, for the model defined by eqn (4), the TOC and BC concentrations together explained 71% of the variation in cumulative average power density (*R*_sq._ = 0.71) and the model using both TOC and BC as predictors fit the power data better than only the mean cumulative average power density (*p* < 0.05, ANOVA). Interestingly, the cumulative average power densities for the 2014 San Diego Bay data were positively correlated (*p* < 0.05, ANOVA) with the percent of TOC and negatively correlated (*p* < 0.05, ANOVA) with the percent of BC found in the sediment.

**Fig. 5:**
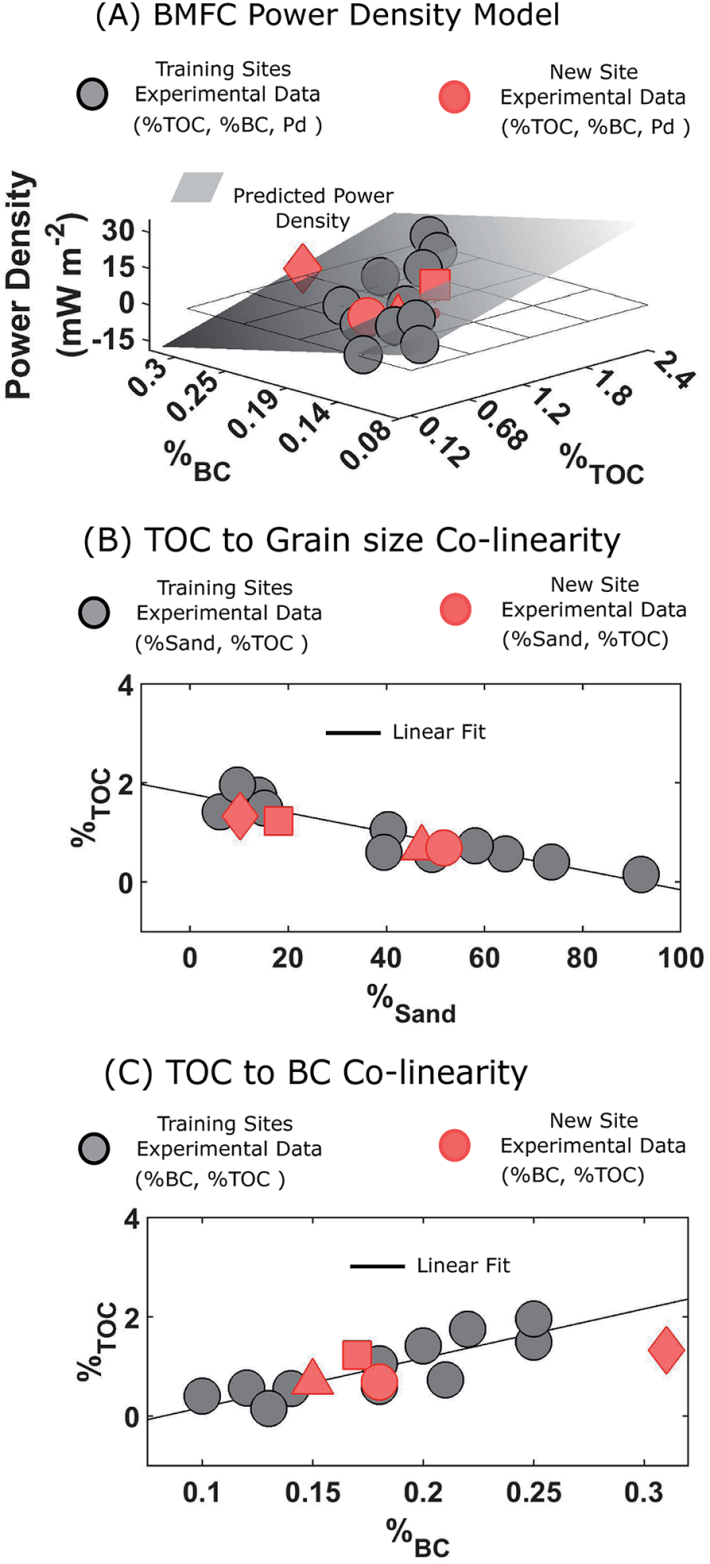
San Diego Bay sediment and sediment-BMFC correlations. (A) Multivariable linear model fit by least squares to BMFC power density and sediment TOC and BC data. The observations are shown as colored markers and the grey plane indicates the least squares fit to the initial data. (B) Correlations between sediment TOC and grain size percentages. (C) Correlations between sediment TOC and BC percentages.

Co-linear relationships existed (*p* < 0.05, ANOVA) between TOC, and the grain size and BC concentration in the sediment, according to the simple linear regression models^26^5
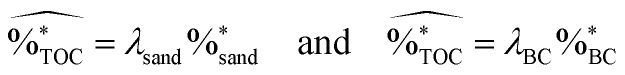
where *λ*_sand_ = −0.92 and *λ*_BC_ = 0.86. Sand concentration explained 85% of the variation in TOC (*R*_sq._ = 0.85) and the negative correlation is consistent with previous hypothesis that high TOC levels tend to be correlated with fine sediments.^7^ The BC concentration explained 74% of the variation in TOC (*R*_sq._ = 0.74) giving a variance inflation factor (=1/1 − *R*_sq._) of only 3.8, thus indicating no multicollinearity problem for the model defined by eqn (4). Hypothesis testing on a subvector of the regression estimates^26^ verified eqn (4) explained more variance in the cumulative average power densities than simply regressing on TOC (*R*_sq._ = 0.42) or BC (*R*_sq._ = 0.08). Fig. 5A–C displays these modeling results and ESI Tables S.3–S.5† contains summaries.

### 3.2 Predicting BMFC performance in San Diego Bay

**Fig. 6:**
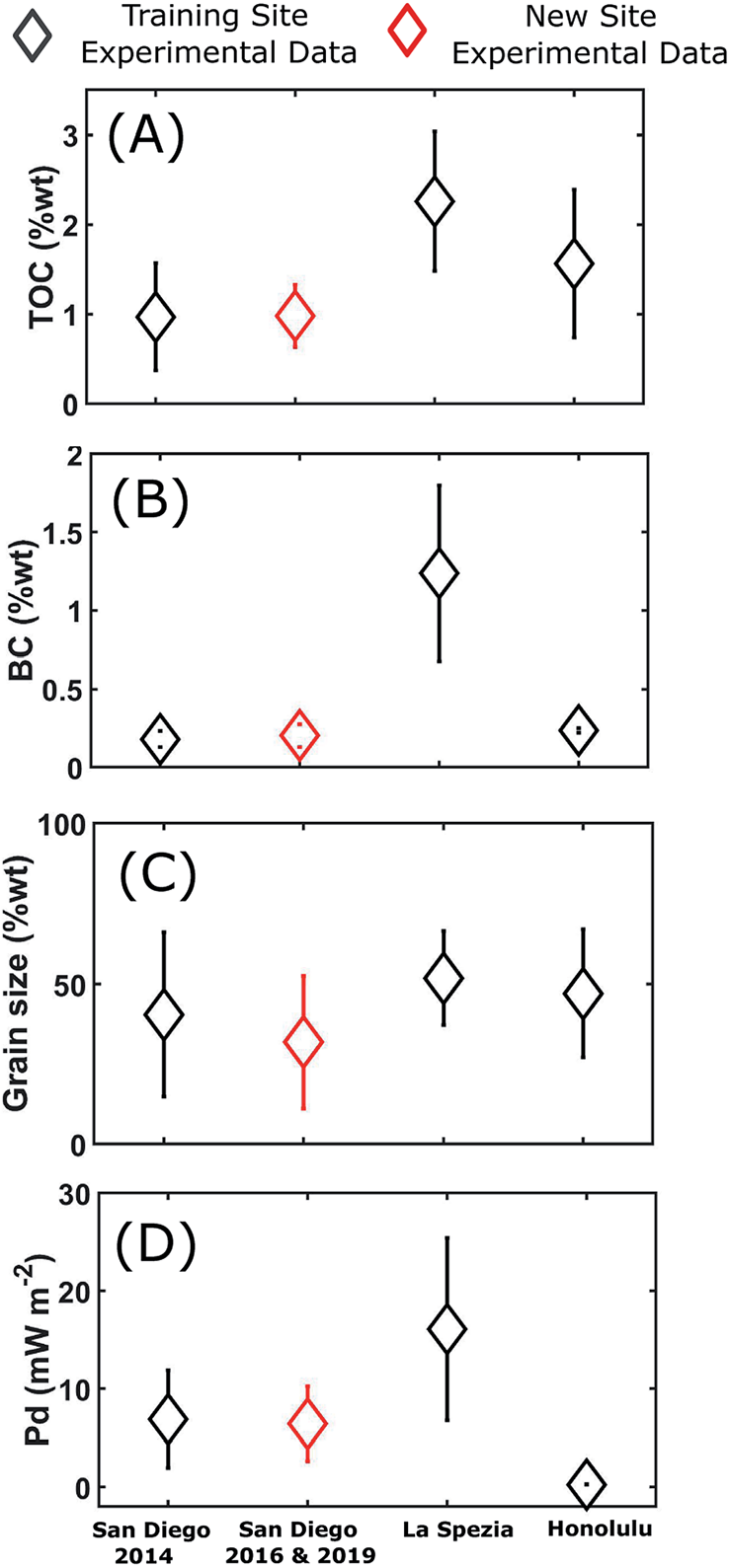
Population averages of San Diego Bay, La Spezia and Honolulu. (A) Total organic carbon: *μ*_TOC_. (B) Black carbon: *μ*_BC_. (C) Grain size: *μ*_sand_. (D) Cumulative average power density *μ*_*P*_d__. Error bars ± SD.

Sediment collection and BMFC experimental data from three San Diego Bay sites (site 1, site 2, site 3) in 2016 and one site (site 11) in 2019 facilitated “New Sites” (see Fig. 1) whose BMFC power performance could be predicted using eqn (4) and compared with actual experimental data. Analysis of the average power density curves, 
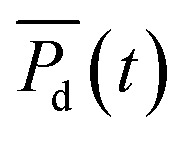
, between the 2014 and later experiments in 2016 and 2019 (Fig. 4) showed similar power performances for site 1 but significant differences between those conducted for sites 2,3 and 11 (*p* < 0.05, 95% CI, *t*-test). Sediment TOC and BC data for the four repeated experiments (Table 1) had sample means: *μ*_TOC_ = 0.98 (wt%) and *μ*_BC_ = 0.20 (wt%) with standard deviations *σ*_TOC_ = 0.35 and *σ*_BC_ = 0.07. Thus, the new site data was comparatively similar to the 2014 experiments (Fig. 6) and therefore did not constitute any major extrapolation of the predictive model. The TOC and sand percentages in each of the samples were closely correlated (Fig. 5B) as predicted by eqn (5).

**Table 1:** Regression modeling results. The model defined by eqn (4), trained on the data from the initial San Diego Bay experiments, was used to predict within a 95% confidence interval the cumulative average power density of the repeated experiments in San Diego Bay and experiments in La Spezia, Italy and Honolulu, Hawaii

	Sample	TOC (wt%)	BC (wt%)	Sand (wt%)	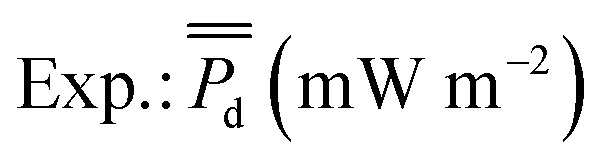	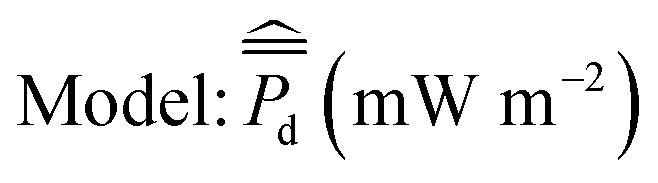
San Diego Bay (CA, USA)	Site 1	1.23	0.17	18.0	11.8	11.3
	Site 2	0.68	0.15	47.2	6.23	6.25
	Site 3	0.68	0.18	51.7	3.89	3.20
	Site 11	1.33	0.31	10.3	0.89	−1.67
La Spezia (Italy)	ITBG 1	2.13	1.66	65.4	6.08	10.0
	ITBG 2	1.37	1.71	61.2	10.9	7.57
	ITSM 1	3.26	1.06	33.0	23.7	20.8
	ITSM 2	2.29	0.52	47.7	25.8	26.0
Honolulu (Hawaii, USA)	HIWA 1	1.67	0.22	33.3	0.28	0.25
	HIWA 2	2.33	0.24	37.8	0.31	0.29
	HIHI	0.69	0.25	70.0	0.26	0.29

The TOC and BC predictors in eqn (4) were standardized using the population means and standard deviations from the 2014 data. The regression coefficients were as trained on the San Diego Bay 2014 data (*i.e. B*_TOC_ = 1.55 and *B*_BC_ = −1.06). Fig. 5A shows, for all four sites laboratory experiments, the cumulative average power densities fell near the plane defined by eqn (4) indicating a good predictive quality of the power performance model. The BMFC power performance from the laboratory experiments and the model for the San Diego Bay locations in 2016 and 2019 is shown in Table 1. The results show, although the power curves were significantly different between several of the 2014 and follow on experiments, BMFC power performance could be accurately forecasted (*R*_sq._ = 0.90) using the TOC and BC characteristics of the surrounding sediment and a trained model.

As a check to rule out variations in the experimental setup between experiments, it was verified the anodes and cathodes for all reactors and all experiments where the same distance apart. Since the geochemical and grain size data of the sediment so closely fit the initial model in eqn (5), anomalies in the laboratory measurements of the replicate data were ruled out. For all four sites, the C : N ratios were similar between the initial 2014, 2016 and 2019 BMFC experiments (see ESI Table S.2†) and grain size was well correlated with TOC (see Fig. 5A). However, there were different TOC and BC content between the two and three year time frames of the initial and follow on experiments. Therefore, it was concluded the variability of power production observed at the replicated site data was attributed to bacterial distribution, their decomposition rates of the organic carbon, and the content of black carbon present in the sediments as predicted by the model.

### 3.3 Extending the modeling paradigm to other regions: La Spezia and Honolulu

The results of the San Diego Bay analysis indicated large-scale site monitoring is essential for identifying trends and drivers of BMFC power performance across a particular region of interest. However, this is highly challenging and costly in terms of coordination, labor and funding – particularly in marine littorals, where access to field sites is difficult and working conditions are harsh. Thus, it was of great interest if the modeling paradigm could capture the variation in BMFC performance, 
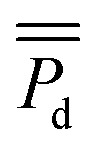
, at two sites in La Spezia (ITBG and ITSM) and two sites in Honolulu (HIWA and HIHI) based on their respective TOC and BC feature vectors. For the interested reader, ESI Fig. S.2† depicts power curves for La Spezia (panels A and B) and Honolulu (panels C and D).

The La Spezia sites were only approximately 3 meters from each other, but their sediment characteristics were quite varied (Table 1). One site (ITBG) was close to a seawall (about 3 meters on either side) and had detritus from bushes and trees. The other site (ITSM) was closer to the La Spezia Bay and had less detritus. The sediment samples, ITBG 1, ITBG 2, ITSM 1 and ITSM 2, had on average higher levels of TOC and BC and were sandier than the San Diego Bay sites (Fig. 6). For the La Spezia sites: *μ*_TOC_ = 2.26 (wt%) and *μ*_BC_ = 1.24 (wt%) with standard deviations *σ*_TOC_ = 0.78 and *σ*_BC_ = 0.56. Therefore, it was concluded the La Spezia sediment data would have resulted in a significant extrapolation of the BMFC power performance model, and its parameters were recomputed. The BMFC population mean cumulative average power density from the La Spezia sediments was much higher than that from San Diego Bay with mean *μ*_*P*_d__ = 16.1 mW m^−2^ and standard deviation *σ*_*P*_d__ = 9.31 (Fig. 6). Using eqn (1) to standardize the La Spezia TOC and BC concentrations and fitting eqn (4) for *B*_TOC_ = 0.15 and *B*_BC_ = −0.64 explained 89% of the variation in cumulative average power density (*R*_sq._ = 0.89). Although it should be noted the data size consisted of only four samples, the results indicated a positive increase in the mean response with respect to TOC and a negative increase with respect to the BC predictor variables.

The first Hawaii sample, HIWA 1, was 14.0 meters deep and located approximately 185 meters from Waipi'o point; situated in the continuously dredged channel entering Pearl Harbor. The second Hawaii sample, HIWA 2, was 12.2 meters deep and was located approximately 13 meters away from a pier. Both samples consisted of very fine unconsolidated sediment. The third Honolulu site HIHI, was situated close to Hickam Airfield. These sites were found to have, on average, higher levels of TOC and were more sandy than the San Diego Bay sites, but with similar concentrations of BC (Fig. 6). For the Honolulu sites: *μ*_TOC_ = 1.56 (wt%) and *μ*_BC_ = 0.24 (wt%) with standard deviations *σ*_TOC_ = 0.83 and *σ*_BC_ = 0.02. The BMFC population mean cumulative average power density from the Honolulu sediments was very low with respect to the San Diego Bay sediments with *μ*_*P*_d__ = 0.29 mW m^−2^ with standard deviation *σ*_*P*_d__ = 0.03 (Fig. 6). As shown in Table 1, repeating the standardization procedure and solving eqn (4) gave *B*_TOC_ = 0.28 and *B*_BC_ = 0.84 which explained 100% of the variation in cumulative average power density (*R*_sq._ = 1.00), but importantly, the data size consisted of only three samples. It remains unknown why such low power performance was observed for the Honolulu samples although very high C : N ratios ∼100 was recorded for the samples.

### 3.4 Impact of sediment parameters on BMFC performance

The investigations of this study found TOC and BC to be the most significant predictors of BMFC power performance. Multivariate analysis on the San Diego Bay region revealed (*p* < 0.05) TOC levels were positively related (*B*_TOC_ > 0) to power performance while BC levels were negatively related to power performance (*B*_BC_ < 0). These observed relationships were also expressed for the La Spezia region. In regards to the contribution of TOC, it is noted bacteria are needed to act as the biocatalysts for generating BMFC power from marine sediment, but their growth rate (and thereby BMFC power production) can be affected by organic carbon availability.^10^ Therefore, fluctuations of TOC drive likewise fluctuations in BMFC cumulative average power density. Additionally, the results of this study suggest for some environments, black carbon also plays a role in limiting BMFC power output. These results are especially interesting in light of other findings in the literature which have shown several types of black carbons found in the environment, such as activated carbon, graphite powder, chars, and soot are chemically active in specific oxidation and reduction reactions^31–33^ which are the driving mechanism of BMFC power production. However, the different magnitude of *B*'s between the San Diego (*B*_TOC_ = 1.55 and *B*_BC_ = −1.06) and La Spezia (*B*_TOC_ = 0.28 and *B*_BC_ = −0.84) regions suggest for BMFC's deployed in vastly different oceanographic environments, the extent to which TOC and BC contribute to BMFC power performance may be different.

**Fig. 7:**
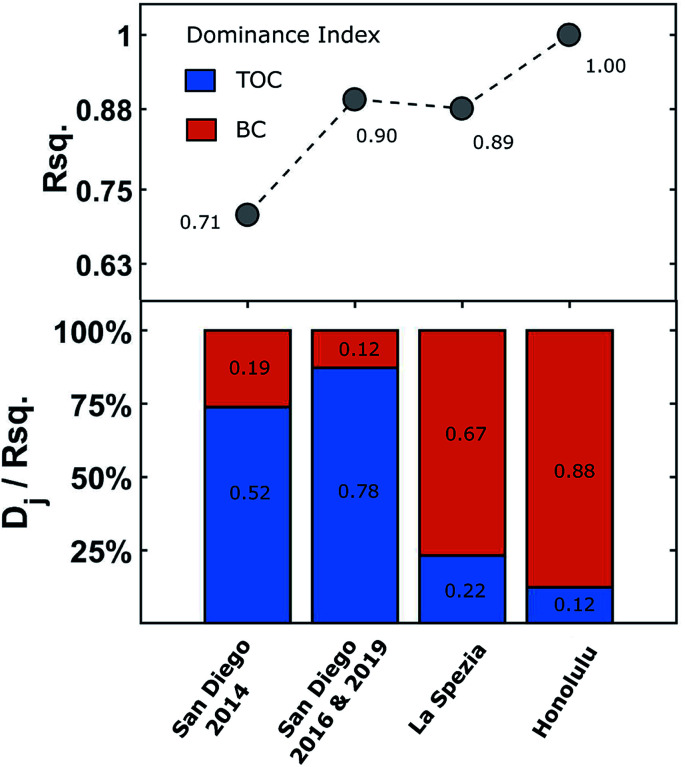
Dominance indices of regression parameters (TOC and BC) contribution on BMFC cumulative average power density: 
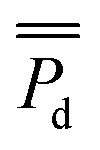
.

To better illustrate the TOC and BC contribution phenomena, the relative contribution of TOC and BC were estimated using eqn (3) to rank the TOC and BC contribution for explaining BMFC power performance based on their dominance indices, *D*_TOC_ and *D*_BC_ respectively. The dominance indices for the San Diego Bay, La Spezia and Honolulu regression models is shown in Fig. 7 along with the associated BMFC power performance model's *R*-squared values. For San Diego Bay, TOC explained 0.52/0.71 = 73% to 0.78/0.90 = 87% of the total variance in BMFC performance, 
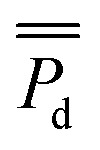
. Including BC into the multivariate model further explained 27% to 13% more variance than TOC alone. A similar analysis on the La Spezia results show BC explained 75% more variance than TOC alone. Hence, the negative impact of BC on BMFC power performance was more pronounced in the La Spezia sediments than those from San Diego Bay.

## 4 Conclusions

This study addressed the *in situ* predictability, expansion of research geography and the impact of sediment parameters on BMFC power performance research. The natural environment presents a host of variables which may affect BMFC performance in intercorrelated and unknown ways. By considering an array of parameters (TOC, BC, C : N and grain size) this study isolated two parameters (TOC and BC) as significant contributors to BMFC performance in terms of BMFC cumulative average power density. Sampling campaigns were conducted over a broad area of interest in San Diego Bay (CA, USA) and methods were extended to La Spezia (Italy) and Honolulu (HI, USA). Importantly, multivariate modeling successfully generated parameters (*B*'s) to capture the impact of TOC and BC on BMFC power performance across spatially and temporally separated sediment samples. Dominance analysis was utilized to illustrate the negative impact of BC on BMFC power performance.

Multivarate analysis proved to be a valuable tool to be employed by research scientists and engineers to predict large scale BMFC performance in diverse littoral environments and facilitate optimal sensor placement. However, the observed dissimilarities of organic carbon contributions across the San Diego Bay, La Spezia and Honolulu regions (Fig. 7) may indicate shifts in the diversity of the bacteria comprising the anode biofilm or an adaptation of the microbial population. Hence, accurate BMFC prediction requires regionally specific modeling. The observed higher levels of BC inhibited or, at the very least, restricted optimal BMFC power performance of in marine sediments. It remains unknown what types of black carbon where encountered between the San Diego Bay, La Spezia and Honolulu sampling territories. More so, the precise mechanism for how black carbon effects BMFC power production (as an adsorbent, electron acceptor or otherwise). Thus, the effects of black carbon on BMFC performance should remain an interesting topic of further research.

As a final remark, while the power densities produced in the laboratory for this study are consistent with those from previous *in situ* studies,^8,10,29^ it is important to note that in the natural coastal aquatic environment, daily variations in temperature, salinity, and water velocity can introduce variations in BMFC power performance^8,10,29^ not captured in the laboratory. Therefore, the laboratory results of this study should represent typical BMFC performance in the field with variations due to contributions from naturally occurring variables.

## Conflicts of interest

There are no conflicts to declare.

## Supplementary Material

RA-010-D0RA03459B-s001
