# Supplementary material for: Impact of sediment parameters in the prediction of benthic microbial fuel cell performance
Source: RSC Adv. 2020 Jul 10;10(44):26220–8. doi: 10.1039/d0ra03459b (PMC9055322; doi:10.1039/d0ra03459b)
Supplement: RA-010-D0RA03459B-s001 [file RA-010-D0RA03459B-s001.pdf]

Electronic Supplementary Material (ESI) for RSC Advances.
This journal is © The Royal Society of Chemistry 2020

Supplementary Information:

Impact of Sediment Parameters in the Prediction of Benthic Microbial Fuel Cell Performance

K.L. Joiner[1,*], G.L. Tukeman[1,2], A.Y. Obraztsova[1], and Y.M. Arias-Thode[1]

[1]Naval Information Warfare Center, San Diego, CA 92152, USA

[2]Center for Precision Environmental Health, Baylor College of Medicine, Houston, Texas

[*]kevin.l.joiner1@navy.mil

Supplementary Table S.1: Locations of sediment sampling sites in San Diego Bay, La Spezia, Italy and Honolulu, Hawaii.

**Location**	**Sample**	**Description**	**Latitude**	**Longitude**
San Diego Bay	Site 1	Sweetwater Channel	32.64891	-117.11285
	Site 2	Channel off Naval Base SD	32.68292	-117.14355
	Site 3	South Bay	32.6298	-117.11960
	Site 4	Scripps Pier	32.70270	-117.23268
	Site 5	Pier 169	32.70537	-117.23586
	Site 6	Fiddlers Cove	32.65600	-117.14389
	Site 7	Glorietta Bay	32.67707	-117.16527
	SD 8	Americas Cup Harbor	32.72320	-117.22378
	Site 9	East Basin	32.72218	-117.17572
	Site 10	South of Ballist Point	32.68046	-117.23316
	Site 11	Marine Corps Recruit Depot	32.73880	-117.20840
La Spezia, Italy	IT 1	Small Grid	44.0959	9.8622
	IT 2	Big Grid	44.0958	9.8620
Honolulu, Hawaii	HI 1	Waipi'o	21.342664	-157.96976
	HI 2	Hickam Airfield	21.32934	-157.9676

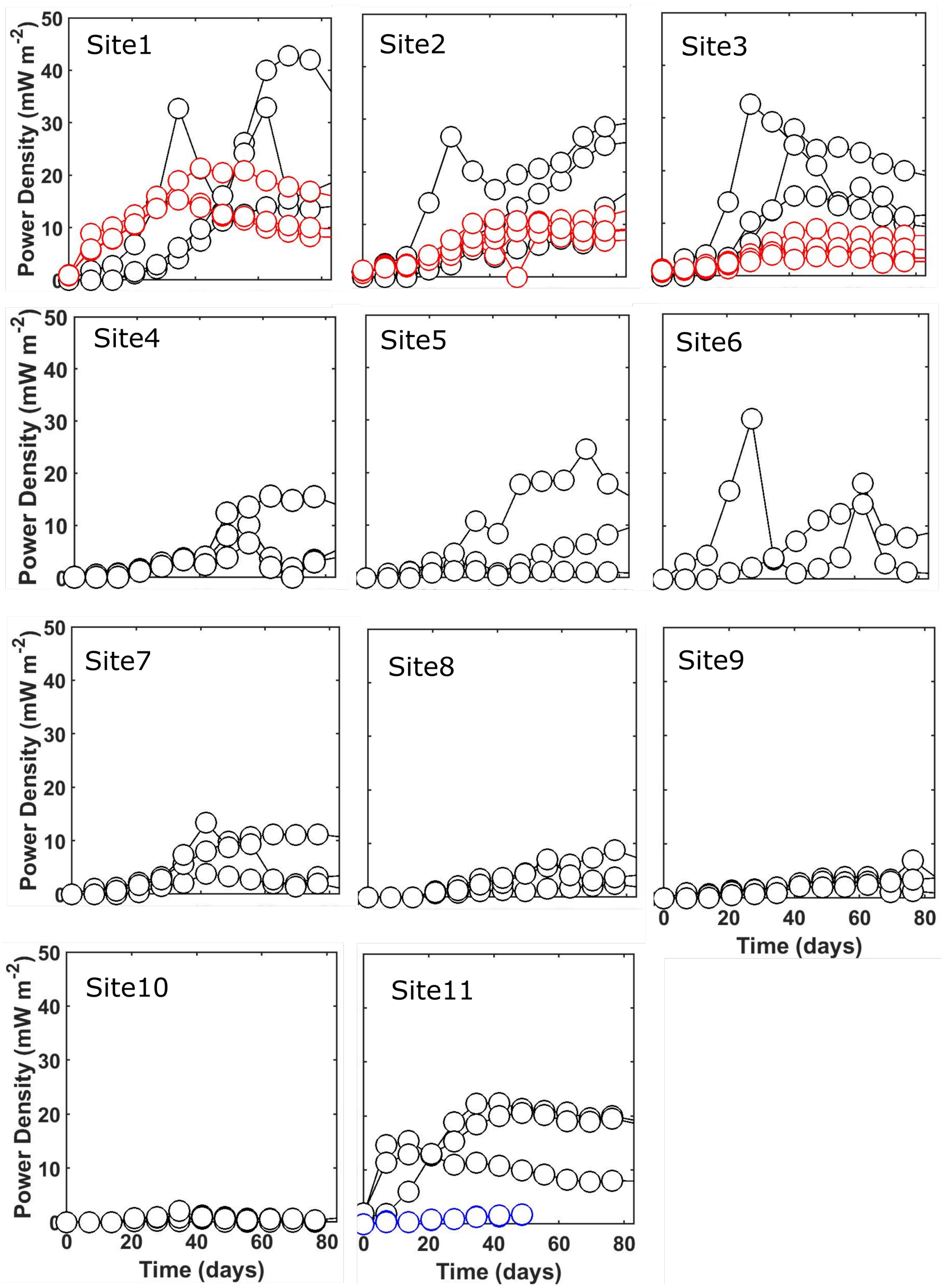

Supplementary Figure S.1: Power density data from the 11 sampling sites in San Diego Bay. Repeated sediment samples where taken in Aug 2014 (black), Sep 2016 (red) and Sep 2019 (blue).

Supplementary Table S.2: Organic geochemical data, textural analysis and average power density from the initial San Diego Bay field samples in 2014 and repeated experiments in 2015 (sites 1-3) and 2019 (site 11). Values entered as (*) denote missing data.

**San D.Bay**	**Sample**	**TOC** (wt%)	**BC** (wt%)	**C : N**	**Sand (wt%)**	**Silt & Clay (wt%)**	**Power :** $\overline{\overline{P_d}}$ **(mW m^{-2})**
Initial Exp.	Site 1	1.75	0.22	12.1	12.4	78.2	13.3
(2014)	Site 2	0.56	0.12	10.2	59.9	33.3	11.4
	Site 3	1.41	0.20	9.13	20.6	82.8	12.7
	Site 4	0.73	0.21	9.55	49.5	41.4	4.36
	Site 5	0.40	0.10	11.0	65.2	27.1	5.06
	Site 6	0.55	0.14	9.33	52.0	42.8	6.36
	Site 7	1.05	0.18	10.2	41.8	53.5	4.13
	Site 8	1.49	0.25	10.5	12.6	82.7	2.52
	Site 9	0.60	0.18	26.0	40.3	51.6	1.94
	Site 10	0.15	0.13	15.5	86.4	11.4	0.38
	Site 11	1.96	0.25	15.8	4.15	94.1	14.0
Repeat Exp.	Site 1	1.23	0.17	10.3	18.0	82.0	11.8
(2016)	Site 2	0.68	0.15	7.30	47.2	52.8	6.23
	Site 3	0.68	0.18	10.0	51.7	48.3	3.89
(2019)	Site 11	1.33	0.31	*	10.25	81.5	0.89

Supplementary Table S.3: BMFC Power density regression analysis using standardized values of sediment TOC and BC. Root mean squared error: 0.191 on 8 degrees of freedom. Multiple R-squared of 0.71. F-statistic vs. constant model: 9.77, p-value < 0.05.

Regression Model: $\overline{\overline{\mathbf{P_d}}} \sim \mathbf{TOC^*} + \mathbf{BC^*}$ (San Diego Bay 2014)

Coefficients	Estimate	Std. Error	t-value	p-value
B_{TOC}	1.55	0.37	4.18	< 0.05
B_{BC}	-1.06	0.37	-2.85	< 0.05

Supplementary Table S.4: Regression analysis using the standardized values of sediment TOC and sand. Root mean squared error: 0.41 on 9 degrees of freedom. Multiple R-squared of 0.85. F-statistic vs. constant model: 51.2, p-value < 0.05.

Regression Model: $\mathbf{TOC^*} \sim \mathbf{Sand^*}$ (San Diego Bay 2014)

Coefficients	Estimate	Std. Error	t-value	p-value
λ_{Sand}	-0.92	0.13	-7.16	< 0.05

Supplementary Table S.5: Regression analysis using the standardized values of sediment TOC and BC. Root mean squared error: 0.54 on 9 degrees of freedom. Multiple R-squared of 0.74. F-statistic vs. constant model: 25.2, p-value < 0.05.

Regression Model: $\mathbf{TOC^*} \sim \mathbf{BC^*}$ (San Diego Bay 2014)

Coefficients	Estimate	Std. Error	t-value	p-value
λ_{BC}	0.86	0.17	5.02	< 0.05

Supplementary Table S.6: BMFC Power density regression analysis using standardized values of sediment TOC and BC. Root mean squared error: 0.27 on 1 degree of freedom. Multiple R-squared of 0.88. F-statistic vs. constant model: 3.71, p-value 0.35.

	**Regression Model: $\overline{\overline{\mathbf{P_d}}} \sim \mathbf{TOC^*} + \mathbf{BC^*}$ (La Spezia)**			
Coefficients	Estimate	Std. Error	t-value	p-value
B_{TOC}	0.15	0.31	0.48	0.71
B_{BC}	-0.64	0.31	-2.05	0.29

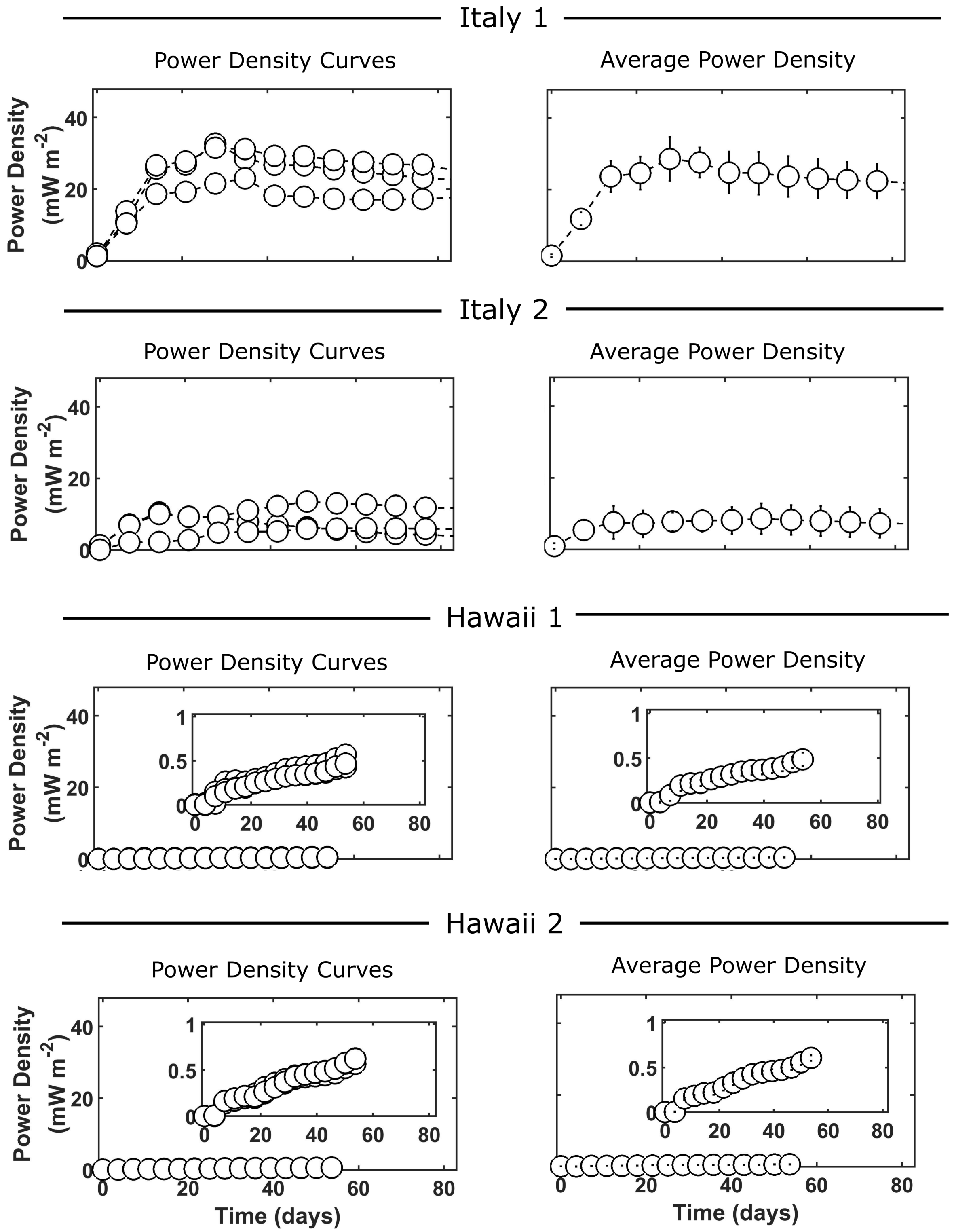

Supplementary Figure S.2: BMFC power density data from sampling sites in Italy and Hawaii in 2016.

Supplementary Table S.7: Organic geochemical data, textural analysis and average power density from the Italy and Hawaii field samples in 2016 and 2019.

	**Sample**	**TOC (wt%)**	**BC (wt%)**	**C : N**	**Sand (wt%)**	**Silt & Clay (wt%)**	**Power :$\overline{\overline{P_d}}$ (mW m^{-2})**
Italy	IT 1	2.78	0.79	25.2	40.35	59.7	21.6
(2016)	IT 2	1.75	1.69	45.0	63.3	36.7	7.04
Hawaii	HI 1	2.00	0.23	91.6	35.6	64.4	0.26
(2019)	HI 2	0.69	0.25	140	70.0	30.0	0.31
